# The potential of targeting autophagy-related non-coding RNAs in the treatment of lung cancer

**DOI:** 10.3389/fphar.2025.1551258

**Published:** 2025-05-14

**Authors:** Juan Li, Jimei Gan, Shenggan Shi, Juying Huang, Yong Yang

**Affiliations:** ^1^ College of Pharmacy, Southwest Medical University, Luzhou, China; ^2^ Department of Pharmacy, Chengdu Wenjiang District People’s Hospital, Chengdu, Sichuan; ^3^ Department of Pharmacy, Personalized Drug Therapy Key Laboratory of Sichuan Province, Sichuan Academy of Medical Sciences and Sichuan Provincial People’s Hospital, School of Medicine, University of Electronic Science and Technology of China, Chengdu, Sichuan, China

**Keywords:** lung cancer, autophagy, non-coding RNA, therapeutic target, chemoresistance

## Abstract

Lung cancer is the most prevalent malignant tumor worldwide and remains the leading cause of cancer-related mortality. Despite advances in treatment development, lung cancer patients often face poor quality of life and low survival rates. Increasing evidence highlights the significant roles of autophagy and non-coding RNAs (ncRNAs) in the initiation, progression, and therapeutic response of lung cancer. Autophagy and ncRNAs can function as both tumor-promoting and tumor-suppressing factors in lung cancer. Therefore, investigating the roles of autophagy and ncRNAs in lung cancer provides valuable insights into its pathophysiology. At the same time, non-coding RNA also plays an important role in regulating autophagy. This study reveals that autophagy affects the occurrence and development of lung cancer through multiple pathways. Then, we also studied that in lung cancer, ncRNAs (e.g., lncRNAs, miRNAs, circRNAs and piRNAs) can regulate autophagy to promote or inhibit tumorigenesis, metastasis and drug resistance in lung cancer. Finally, the problems and solutions of autophagy and ncRNAs in the treatment of lung cancer were explored. These findings suggest that autophagy and ncRNAs can be potential targets for the treatment of lung cancer.

## 1 Introduction

Lung cancer is a malignant tumor that originates in the bronchial mucosa or lung glands. It is one of the most prevalent cancers worldwide (Li Y. et al., 2023). Based on histopathological characteristics, lung cancer is primarily categorized into non-small cell lung cancer (NSCLC) and small cell lung cancer. NSCLC, which includes adenocarcinoma and squamous cell carcinoma [with adenocarcinoma being the most common, followed by squamous cell carcinoma (Abdelaziz et al., 2018)], is the most common subtype, accounting for 85%–90% of all lung cancer cases. In 2020, there are an estimated 2.2 million new cases of lung cancer and 1.8 million deaths from lung cancer (Leiter et al., 2023). Currently, lung cancer ranks as the second most common cancer globally in terms of incidence, but it is the leading cause of cancer-related mortality (Siegel et al., 2023). Globally, lung cancer is the primary cause of cancer death among men and the second among women, following breast cancer (Leiter et al., 2023). The main clinical symptoms are cough, expectoration, hemoptysis, asthma, chest pain, dyspnea and so on. Current treatment options for lung cancer commonly include surgery, radiation therapy, and chemotherapy. The prognosis varies depending on factors such as tumor type, stage, patient age, and gender; however, overall, the five-year survival rate for lung cancer remains low. In terms of prevention, key measures include reducing smoking, avoiding occupational and environmental exposure, and undergoing regular health screenings. Recently, there is growing evidence that autophagy may specifically regulate lung cancer through regulating autophagy-related genes expression and some signaling pathways (Liu et al., 2017).

**FIGURE 1:**
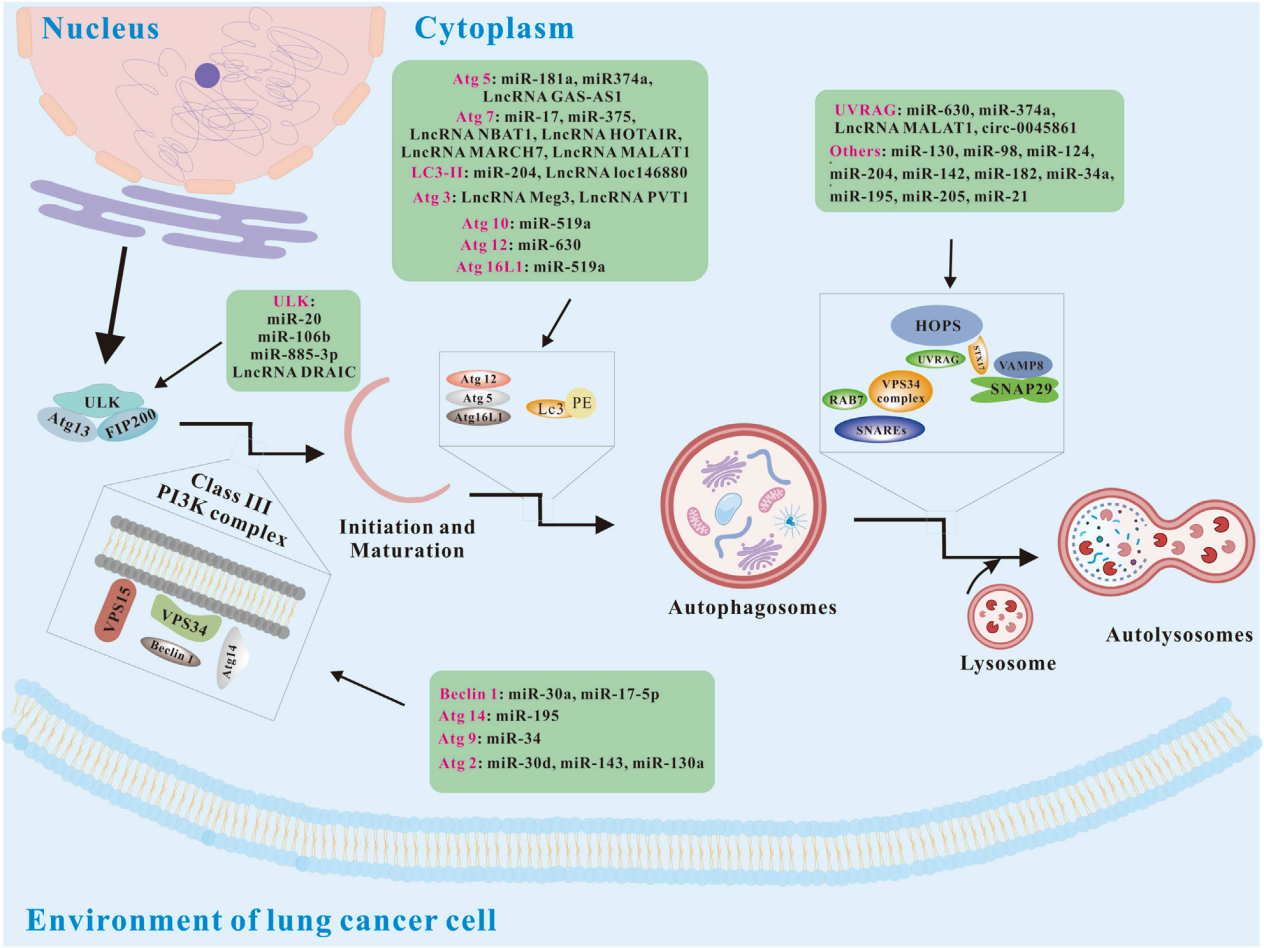
Basic autophagy pathways and modulation via ncRNAs (modified from (Rezaei et al., 2020)). Atg 2, autophagy-related 5; Atg 5, autophagy-related 5; Atg 9, autophagy-related 9; Atg 10, autophagy-related 10; Atg 12, autophagy-related 12; Atg 13, autophagy-related 13; Atg14, autophagy-related 14; Atg 16L1, autophagy-related 16 like 1; FIP200, focal adhesion kinase family interacting protein of 200; HOPS, homotypic fusion and protein sorting; LC3, Microtubule-associated protein 1 light chain 3; PI3K, phosphatidylinositol 3-kinase; STX17, syntaxin 17; SNAP29, synaptosome-associated protein 29; SNAREs, soluble N-ethylmaleimide-sensitive factor attachment protein receptors; ULK, unc-51-like kinase; UVRAG, UV radiation resistance associated; VAMP8, vesicle-associated membrane proteins 8; VPS 15, vesicular protein sorting 15; VPS34, vesicular protein sorting 34.

Autophagy is a highly conserved catabolic process in eukaryotic cells, mediated by autophagy-related genes (Atgs) (Levine and Kroemer, 2019). Alongside the proteasome system, it is one of the cell’s two primary degradation pathways. Fundamentally, autophagy functions to clear damaged or unnecessary organelles, invading pathogens, and misfolded proteins, helping to maintain cellular homeostasis and supply energy (Nemchenko et al., 2011; Wang et al., 2013). Recently, it has also been linked to health maintenance and longevity (Levine and Kroemer, 2008). There are at least three major types of autophagy including macro-autophagy (hereafter referred to as autophagy), micro-autophagy, and chaperone-mediated autophagy (CMA). Autophagy consists of four basic steps: 1) the initial stage induced by nutrient restriction or removal of damaged or redundant organelles; 2) the formation of bilayer membrane-bound autophagosomes encapsulating organelles, proteins and cytoplasm; 3) the formation stage of autolysosome; 4) the autolysosome cargo digestion and renewal. The above autophagy process is regulated by multiple autophagy-related proteins, including the unc-51-like kinase 1 complex (ULK1C), phosphatidylinositol-3-phosphate (PI3P), double FYVE domain protein 1 (DFCP1), and phosphatidylinositol-interacting WD repeat protein (WIPI), which are crucial for autophagosome formation. Microtubule-associated protein 1 light chain 3 (MAP1LC3) is involved in autophagosome maturation, while autophagic lysosome formation is associated with the homotypic fusion and protein sorting (HOPS) complex, syntaxin 17 (STX 17), vesicle-associated membrane proteins 7 and 8 (VAMP 7/8), and synaptosome-associated protein 29 (SNAP 29). Studies have shown that many non-coding RNAs can regulate autophagy (Figure 1).

Non-coding RNA (ncRNA) refers to RNA molecules transcribed from the genome that do not encode proteins (Slack and Chinnaiyan, 2019). ncRNAs can be broadly divided into two main types: basic structural ncRNAs and regulatory ncRNAs. Within regulatory ncRNAs, these molecules are further categorized based on their length, structure, and location. The four primary types of regulatory ncRNAs involved in cancer include microRNA (miRNA), long non-coding RNA (lncRNA), circular RNA (circRNA), and PIWI-interacting RNA (piRNA) (Hon et al., 2021). LncRNAs and circRNAs are both over 200 nucleotides in length; however, lncRNAs are linear, whereas circRNAs are circular (Hon et al., 2021). LncRNAs play key roles in various biological processes, such as epigenetic regulation, cell cycle control, and cell differentiation. CircRNAs serve multiple functions as well, acting as miRNA sponges, transcriptional regulators, and binding partners for RNA-binding proteins (Zhao et al., 2014). miRNAs are single-stranded molecules approximately 20–24 nucleotides long and regulate post-transcriptional gene expression by pairing with complementary sequences in the 3′UTR of target mRNA transcript (Vos et al., 2019). PiRNAs, ranging from about 24 to 31 nucleotides, are primarily found in mammalian germ cells and stem cells. By binding to Piwi proteins to form piRNA complexes, they regulate gene silencing pathways (Zeng et al., 2020).

**FIGURE 2:**
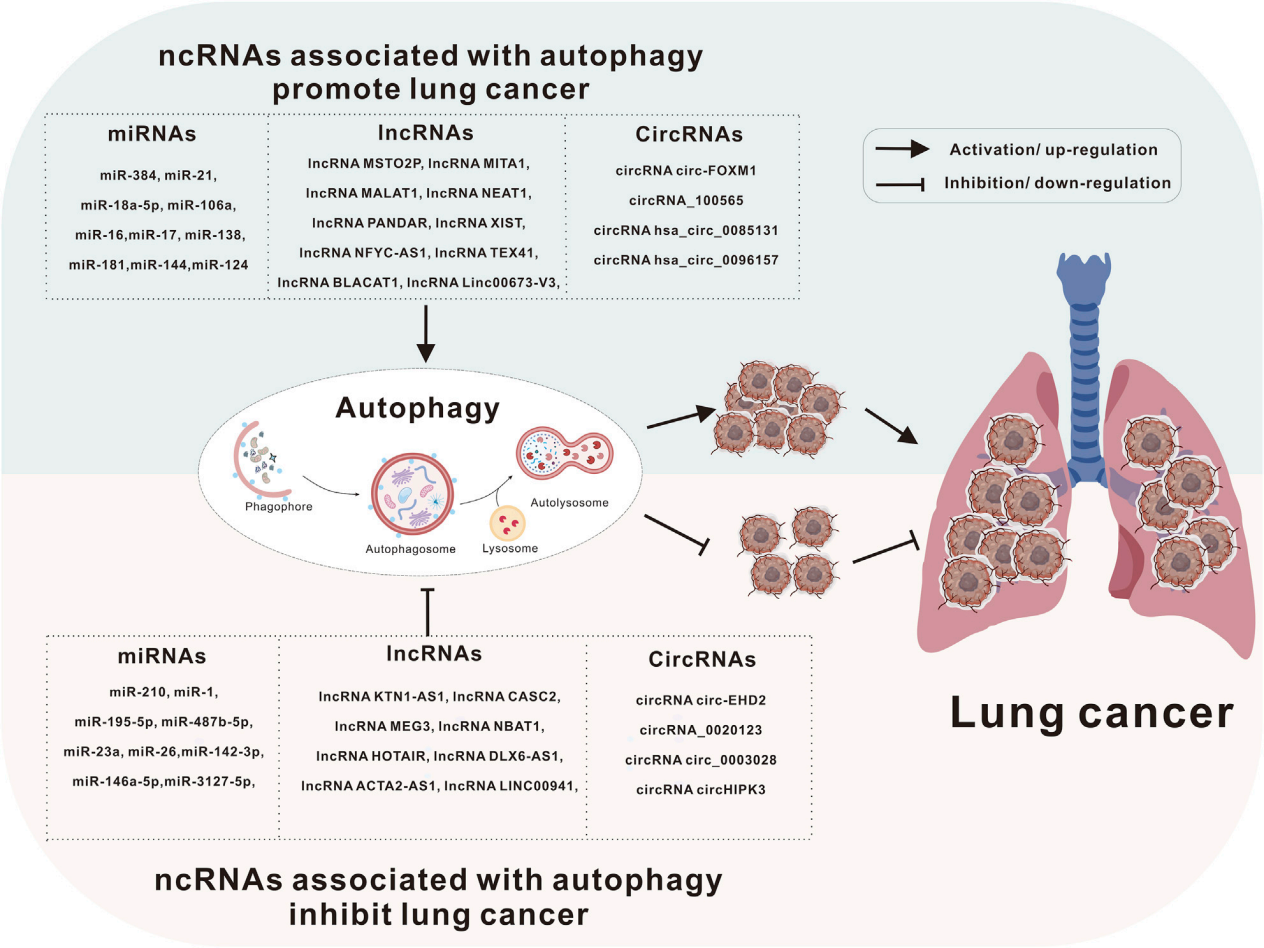
Autophagy and ncRNAs are involved in the development of lung cancer. Lung cancer can be caused by a variety of causes, including heredity, long-term inhalation of toxic substances (e.g., smoking), and even some treatment methods may also be factors leading to lung cancer. Autophagy and ncRNAs are involved in the pathological process of lung cancer through different mechanisms.

A substantial body of evidence shows that ncRNAs and autophagy play critical roles in human malignancies. ncRNAs can function as either oncogenes or tumor suppressors, influencing cancer initiation and progression, while autophagy can similarly act as a tumor suppressor or pro-cancer factor in regulating cancer development. Moreover, many ncRNAs can be released from cancer cells into the blood or urine, where they serve as diagnostic markers or prognostic indicators. Notably, these ncRNAs, acting as tumor markers, may have a close relationship with autophagy. This article reviews recent advances in understanding the roles of these key ncRNAs and autophagy in lung cancer (Figure 2).

## 2 Autophagy in lung cancer

A number of studies have shown that autophagy is closely related to the occurrence, development and prognosis of lung cancer. Although the role of autophagy in lung cancer cells needs to be further evaluated, it seems to be involved in the occurrence of lung cancer and plays a dual role of promotion or inhibition at different stages of lung cancer development (Chen L. et al., 2019; Chen P. et al., 2019; Guo et al., 2019; Ju et al., 2019).

### 2.1 Autophagy-related genes in lung cancer

Autophagy-related genes play a crucial role in the formation of autophagosomes (Shi and Jiang, 2022). Additionally, abnormal expression of *Atgs* may be an important factor affecting lung cancer. Recently, Kim et al. found that USP15 interacted with BECN1 and induced deubiquitination of BECN1, thereby attenuating autophagy induction and negatively regulating lung cancer progression (Kim et al., 2022). Cai et al. reported that casein kinase 1 alpha 1 (CK1α) acted as an autophagy inducer to activate autophagy regulation and inhibited tumor growth through the PTEN/AKT/FOXO3a/Atg7 axis. It is worth noting that blocking CK1α-induced Atg7-dependent autophagy and carcinogenic HRasV12 synergistically initiate tumorigenesis of lung epithelial cells (Cai et al., 2018). Guo et al. showed that Overexpression of the *TSTA3* gene promoted the malignant characteristics of lung squamous cell carcinoma by regulating LAMP2-mediated autophagy and tumor microenvironment (Guo et al., 2023). Zhang et al. reported that miRNA-153-3p inhibited autophagy and promoted gefitinib sensitivity in non-small cell lung cancer by inhibiting the expression of autophagy-related gene *ATG5* (Zhang W. et al., 2019). These studies provided direct evidence that abnormal expression of autophagy-related genes was closely related to the occurrence and progression of lung cancer.

### 2.2 Autophagy-related signaling pathway in lung cancer

#### 2.2.1 mTOR-related signaling pathway

Mechanistic target of rapamycin (mTOR) is a major regulator of cell growth and metabolism, promoting anabolic processes such as ribosome biogenesis and the synthesis of proteins, nucleotides and lipids, and inhibiting catabolic processes such as autophagy. Dysregulation of mTOR signaling is associated with many human diseases, including diabetes, neurodegenerative diseases and cancer (lung cancer) (Shimobayashi and Hall, 2014). PI3K-AKT-mTOR signaling pathway, as a classic insulin signaling pathway, participates in the occurrence and development of various diseases by mediating autophagy. For instance, Di et al. found that ailanthone enhanced cisplatin-induced apoptosis and autophagy in NSCLS through the PI3K/AKT/mTOR pathway (Di et al., 2024). Prucalopride, a dihydrobenzofuran formamide compound and highly selective 5-HT4 receptor agonist, was reported by Chen et al. to inhibit the proliferation, invasion, and migration of lung cancer cells by blocking the PI3K/Akt/mTOR signaling pathway (Chen M. et al., 2019). Xu et al. identified C-C motif ligand 2 (CCL2), a well-known CC chemokine involved in tumor progression, as a promoter of metastasis and epithelial–mesenchymal transition in non-small cell lung cancer through the PI3K/Akt/mTOR and autophagy pathways (Xu et al., 2023). Additionally, Luo et al. reported that SLL-1A-16, a novel organoselenium compound, demonstrated anti-proliferative effects on NSCLC by inducing autophagy and inhibiting cell proliferation via the Akt/mTOR pathway (Luo X. et al., 2024). Wang et al. found that CAMK2 inhibitor 1 (CAMK2N1) promoted autophagy and apoptosis through the Akt/mTOR pathway, effectively inhibiting invasion, migration, and angiogenesis in NSCLC. The adenosine monophosphate-activated protein kinase (AMPK)/mTOR signaling pathway is another critical pathway that plays a pivotal role in maintaining energy and redox balance at both cellular and organismal levels (Inoki et al., 2012). Dysregulation of this pathway can disrupt these balances and contribute to tumor development. Ma et al. reported that COTE-1 (also known as FAM189B), which encoded a membrane protein, was widely expressed in various tissues and activated autophagy via the AMPK/mTOR signaling pathway to promote proliferation and invasion in small cell lung cancer (Ma et al., 2023). Zhu et al. found that cycloastragenol induced protective autophagy through the AMPK/ULK1/mTOR pathway and promoted apoptosis in human non-small cell lung cancer cell lines by upregulating NOXA expression (Zhu et al., 2024). Additionally, Luo et al. demonstrated that pseudolaric acid B (PAB) inhibited NSCLC progression through the ROS/AMPK/mTOR/autophagy signaling pathway (Luo D. et al., 2024). Li et al. reported that resveratrol induced autophagy and apoptosis in non-small cell lung cancer cells by activating the NGFR-AMPK-mTOR pathway (Li et al., 2022a). The above studies have shown that mTOR-related signaling pathways are closely related to the occurrence and progression of lung cancer.

#### 2.2.2 EGFR signaling pathway

Epidermal growth factor receptor (EGFR) is a receptor for epithelial growth factor cell proliferation and signal transduction. In the process of organogenesis and tissue repair, the EGFR pathway usually provides a strong signal for epithelial cell proliferation/survival (Goldkorn and Filosto, 2010). EGFR can initiate lung tumorigenesis by activating pro-survival and anti-apoptotic cellular responses, including increased proliferation, motility, angiogenesis, vasculogenic mimicry, and invasiveness (Prenzel N et al., 2001; Levantini et al., 2022). For instance, Wang et al. reported that combination of betulinic acid (BA) and epidermal growth factor receptor tyrosine kinase inhibitors (EGFR-TKIs) exerted a synergistic anti-tumor effect by inducing autophagy-related cell death through the EGFR signaling pathway (Wang et al., 2024). Yu et al. found that blocking EGFR-SQSTM1 interaction with short peptide SAH-EJ2 interference inhibited lung cancer by activating autophagy and inhibiting EGFR signal transduction (Yu et al., 2020). Thyroid hormone receptor interactor 13 (TRIP13) is an ATP enzyme that is overexpressed in a variety of tumors and is involved in tumor drug resistance. Xiao et al. found that TRIP13 overexpression promoted gefitinib resistance in non-small cell lung cancer by regulating autophagy and phosphorylation of EGFR signaling pathway (Xiao et al., 2023).

#### 2.2.3 Others signaling pathway

Recent research found by Guo et al. showed that quercetin induced pro-apoptotic autophagy in human lung cancer cells through the SIRT1/AMPK signaling pathway *in vitro* (Guo et al., 2021). Li et al. reported that programmed death ligand-1 (PD-L1) promotes primary resistance of EGFR-mutant lung adenocarcinoma cells to EGFR-TKIs, potentially by inducing autophagy via the MAPK signaling pathway (Li N. et al., 2024). Additionally, Chen et al. demonstrated that baicalin participates in ferritinophagy and modulates macrophage immunity through the KEAP1-NRF2/HO-1 axis, thereby enhancing NSCLC sensitivity to cisplatin (Chen et al., 2024). Ding et al. reported that regulator of G protein signaling 20 (RGS20) activated autophagy and promoted the proliferation of non-small cell lung cancer by inhibiting PKA-Hippo signaling pathway (Ding et al., 2024). Fan et al. found that bruceine D induced lung cancer cell apoptosis and autophagy via the ROS/MAPK signaling pathway *in vitro* and *in vivo* (Fan et al., 2020). Bai et al. reported that protein disulfide isomerase family 6 (PDIA6), an oncogene, inhibited JNK/c-Jun signaling pathway by interacting with MAP4K1, thereby inhibiting cisplatin-induced apoptosis and autophagy in NSCLC cells (Bai et al., 2019). Zhang et al. found that phanginin R (PR) induced cytoprotective autophagy in NSCLC cells via the JNK/c-Jun signaling pathway, and inhibition of autophagy could further improve the anti-cancer potential of PR (Zhang L. L. et al., 2020). Liu et al. reported that YZT, a novel PDK1/MEK dual inhibitor, induces protective autophagy in non-small cell lung cancer cells through the PDK1/Akt signaling pathway (Liu R. et al., 2023).

### 2.3 Autophagy and NSCLC drug resistance

Autophagy is a crucial survival mechanism under stress, playing a key role in maintaining cell homeostasis and regulating proliferation (Mizushima et al., 2020). In early cancer development, autophagy helps safeguard normal cells from tumorigenesis by preventing DNA damage and mutations. In established solid tumors, autophagy facilitates tumor progression by promoting growth, survival, chemotherapy resistance, and metastasis in response to stressors such as hypoxia, drug exposure, and nutrient deprivation (Mulcahy Levy and Thorburn, 2019; Leonardi et al., 2022). Thus, autophagy plays a dual role in non-small cell lung cancer (NSCLC), influencing both tumor progression and drug resistance.

#### 2.3.1 Autophagy and traditional chemotherapy drug resistance in NSCLC

Chemotherapy remains a primary treatment for NSCLC; however, drug resistance poses a significant challenge. Studies have linked NSCLC chemoresistance to protective autophagy. For instance, Chen et al. found that cisplatin treatment upregulated autophagy-related genes in NSCLC, suggesting that autophagy contributes to cisplatin resistance in A549 cells (Chen et al., 2018). Using autophagy inhibitors, or knocking down the expression of autophagy-related genes or proteins, could enhance the sensitivity of lung cancer cells to cisplatin (Chen J. et al., 2022). Moreover, autophagy strengthens tumor resistance by modulating the tumor microenvironment and immune response (Xia et al., 2021). However, chemotherapy can trigger both protective autophagy and autophagic cell death. Bai et al. reported that esomeprazole inhibited V-ATPase expression, inducing autophagy and leading to paclitaxel (PTX) accumulation in A549/Taxol-resistant cells. This significantly reduced cell proliferation and resistance (Bai et al., 2020). These findings suggest that autophagy’s role in NSCLC chemoresistance varies depending on the drug and tumor cell type. Therefore, targeting autophagy in NSCLC treatment requires careful consideration.

#### 2.3.2 Autophagy and molecular targeted drug resistance in NSCLC

It has been reported that approximately 73.9% of NSCLC patients have driver gene mutations, with EGFR, ALK, and ROS1 being the most common (Sholl et al., 2015; Wen et al., 2019). Recent advancements have led to the development of molecular targeted therapies, including immune checkpoint inhibitors (Liu and Mao, 2024), and tyrosine kinase inhibitors (Dzul Keflee et al., 2022), for NSCLC. These therapies have notably improved the prognosis and extended survival in patients with driver gene-positive NSCLC. However, the benefits are often short-lived, as most patients develop resistance within a year of treatment (Miller et al., 2022). Drug resistance arises from multiple factors, with autophagy playing a significant role. For instance, Li et al. found that osimertinib, a third-generation EGFR-TKI, induces autophagy in NSCLC cells. Increased autophagy correlated with osimertinib resistance in both *in vitro* and *in vivo* studies. Suppressing autophagy enhanced the drug’s cytotoxicity in both resistant and sensitive cells (Li L. et al., 2019). Lu et al. demonstrated that lorlatinib triggered both apoptosis and protective autophagy in ALK-positive NSCLC cells. However, this protective autophagy reduced lorlatinib’s cytotoxic effects on ALK-positive NSCLC cells. Combining lorlatinib with the autophagy inhibitor CQ activated the Foxo3a/Bim axis, suppressing autophagy and enhancing apoptosis in both *in vitro* and *in vivo* models (Lu et al., 2022).

These findings suggest that autophagy significantly contributes to lung cancer development. As such, targeting autophagy with specific modulators could represent a promising therapeutic strategy (Table 1). In summary, tumor cells manipulate autophagy through various signaling pathways to counteract drug toxicity, thus enhancing their survival. Therefore, combining autophagy modulators with targeted therapies may help overcome drug resistance in NSCLC. Nevertheless, further research is needed to precisely control autophagy for therapeutic benefit.

**TABLE 1:** The potential autophagy modulators for treating lung cancer.

Autophagy modulators	Targets	Mechanisms	References
Alpha-hederin	ROS	Autophagy inhibitor α-Hed increased the killing effect of Tax on NSCLC cells by promoting ROS accumulation	Belmehdi et al. (2023)
Andrographolide	STAT3	Andrographolide suppressed NSCLC progression through induction of autophagy and antitumor immune response	Wang et al. (2022a)
Aniline TFPA	ROS	TFPA increases the production of reactive oxygen species by autophagy damage induced by combination with camptothecin, which ultimately leads to apoptosis of lung cancer cells	Chou et al. (2025)
Asiatic acid	PI3K	Asiatic acid impeded NSCLC progression by inhibiting COX-2 and modulating PI3K signaling, leading to an induction of cytotoxic autophagy-mediated apoptosis	Singh et al. (2024)
Baicalein	MAP4K3	Baicalein enhanced the rapid transport of TFEB to the nucleus and triggers TFEB-dependent autophagy by inducing the degradation of MAP4K3, leading to the arrest of lung cancer cell proliferation	Li et al. (2021)
Baicalin	KEAP1-NRF2/HO-1	Baicalin promoted the sensitivity of NSCLC to cisplatin by regulating ferritinophagy and macrophage immunity through the KEAP1-NRF2/HO-1 pathway	Chen et al. (2024)
Betulinic acid	EGFR	Betulinic acid enhanced the inhibitory effect of EGFR-TKIs by blocking EGFR and regulating the EGFR-ATK-mTOR axis, and activated EGFR-induced autophagic cell death	Wang et al. (2024)
Chloroquine	ROS	Chloroquine inhibited paclitaxel resistance and reduced metastatic potential of human non-small cell lung cancer cells through ROS-mediated β-catenin pathway	Datta et al. (2019)
Curcumin	Ulk1	Curcumin sensitizes NSCLC cells to crizotinib by inhibiting autophagy through the regulation of miR-142-5p and its target Ulk1	He et al. (2022)
	PI3K/Akt/mTOR	Curcumin inhibited the cell growth of NSCLC cells through inducing apoptosis and autophagy by inhibition of the PI3K/Akt/mTOR pathway	Liu et al. (2018b)
CUR5g	STX17	CUR5g effectively inhibits autophagy in tumor tissues *in vivo* by inhibiting STX17 and enhances the sensitivity of lung cancer cells to cisplatin	Chen et al. (2022a)
Hibiscus manihot L. flower extract	ATG5/7-dependent autophagy	HML induced apoptosis in A549 cells by activation of the PTEN-P53 pathway and inhibition of ATG5/7-dependent autophagy	Xu et al. (2024)
Metformin	AMPK	Metformin inhibited autophagy and enhanced the sensitivity of osimertinib by inducing AMPK activation	Chen et al. (2019e)
Resveratrol	miR-671-5p/STOML2/PINK1/Parkin-mediated autophagy	Resveratrol inhibited mitophagy through miR-671-5p-mediated STOML2, which increased the susceptibility of A549/paclitaxel cells to paclitaxel	Kong et al. (2023)
	lncRNA ZFAS1/miR-150-5p/PINK1	Resveratrol influenced mitophagy via ZFAS1/miR-150-5p mediated PINK1/Parkin pathway and enhanced the antitumor activity of paclitaxel in NSCLC.	Kong et al. (2022)
Ursodeoxycholic acid	TGF-β/MAPK	Ursodeoxycholic acid inhibited autophagy pathway through TGF-β/MAPK pathway and enhanced the efficacy of doxorubicin in NSCLC	Li et al. (2024b)
Yifei-Sanjie pill	PI3K/Akt/mTOR	YFSJ reduced the resistance of NSCLC to gefitinib (EGFR-TKI) by inhibiting the PI3K/Akt/mTOR pathway and increasing autophagy	Zhang et al. (2025)
YM155	AKT/mTOR	YM155 induced autophagy-dependent apoptosis and autophagic cell death through AKT/mTOR pathway, which significantly enhanced the sensitivity of erlotinib to EGFR-TKI resistant NSCLC cells	Dai et al. (2018)
β-Elemene	Rab7	β-elemene overcomed lncRNA H19-mediated autophagy-induced EGFR degradation by inhibiting Rab7 levels, and then enhanced the sensitivity of lung cancer to gefitinib by relocating EGFR to the plasma membrane	Zhang et al. (2024)

## 3 ncRNAs in lung cancer

### 3.1 MiRNA in LC

#### 3.1.1 miRNAs in the modulation of cell proliferation and metastasis in LC

A growing body of research highlights the critical role of microRNAs (miRNAs) in regulating cell proliferation, tumor metastasis, and angiogenesis. For instance, Gao et al. found that elevated levels of miR-485-5p suppressed tumor cell invasion by targeting the FLOT2 protein, thereby reducing the ability of cancer cells to infiltrate surrounding tissue (Gao et al., 2019). Similarly, Mao et al. reported that exosomal miR-375-3p disrupted the vascular barrier, increasing its permeability and subsequently facilitating cancer cell migration and metastasis (Mao et al., 2021). In addition, Ma et al. observed that lower expression of miR-320b was associated with improved overall survival in lung cancer patients. MiR-320b exerts its effects by targeting hepatocyte nuclear factor 4γ, thereby inhibiting tumor growth, invasion, and angiogenesis in xenograft models (Ma et al., 2021).

#### 3.1.2 MiRNAs related with chemotherapy in LC

Moreover, miRNAs are key regulators of multidrug resistance mechanisms in NSCLC. Shi et al. observed that elevated levels of miR-20a in exosomes from tumor-associated fibroblasts in NSCLC patients, which contributed to cisplatin resistance in NSCLC cells. Inhibition of PTEN, a direct target of miR-20a, was shown to suppress both cell proliferation and chemoresistance in NSCLC (Shi et al., 2022). Hao et al. reported that miR-369-3p, which was overexpressed in NSCLC, directly targeted the SLC35F5 gene. By regulating nucleotide sugar transport, it influenced drug assimilation and ultimately contributed to cisplatin resistance (Hao et al., 2017). Haque et al. identified Sonic Hedgehog (SHH) as a novel target of miR-506-3p, noting its abnormal activation in epidermal growth factor receptor-resistant (ER) cells. Reduced miR-506-3p expression in ER cells enhanced resistance to EGFR-TKIs by modulating SHH signaling, upregulating E-cadherin, and suppressing vimentin (Haque et al., 2020). Wu et al. discovered that low miR-630 expression in EGFR-mutated lung adenocarcinoma conferred TKI resistance via the miR-630/YAP1/ERK feedback loop. As a result, miR-630 may serve as a potential biomarker for predicting treatment response and clinical outcomes in patients receiving TKI therapy. Additional insights into the role of miRNAs in lung cancer are provided in studies by Lobera et al. (2023) and Gilyazova et al. (2023).

### 3.2 LncRNAs in LC

Long non-coding RNAs (lncRNAs) are RNA molecules exceeding 200 nucleotides in length that do not encode proteins (Mercer et al., 2009; Volders et al., 2013). They serve as key regulators in a wide range of biological processes and signaling pathways, and are strongly associated with disease onset and progression. While the roles of certain lncRNAs are well-characterized, the functions of most remain largely unknown. Recent research indicates that aberrant lncRNA expression contributes to tumor development, invasion, and drug resistance through various mechanisms across different cancer types (Huarte, 2015; Lin and Yang, 2018). Uncovering new molecular pathways involving lncRNAs may offer promising therapeutic targets and enhance our understanding of lung cancer pathogenesis and its intricate regulatory networks (Ding et al., 2018; Bader et al., 2011; Hayes et al., 2014; Tang et al., 2017; Tian et al., 2017; Yang et al., 2014).

#### 3.2.1 LncRNAs related with chemotherapy in LC

Among lncRNA-related research, the majority focuses on their involvement in chemotherapy response. One mechanism involves direct regulation of downstream genes by lncRNAs, which alters lung cancer chemosensitivity. For instance, Xu et al. showed that lncRNA XIST bound to SMAD2, inhibiting its nuclear translocation. This suppressed SMAD2-induced transcription of p53 and NLRP3, thereby reducing cisplatin (DDP)-induced pyroptosis and decreasing chemosensitivity (Xu et al., 2020). Similarly, elevated levels of lncRNA UCA1 have been linked to cisplatin resistance in NSCLC patients (Li C. et al., 2019). In addition, lncRNAs contributed to drug resistance by acting within the competitive endogenous RNA (ceRNA) network. Ge et al. reported that lncRNA SNHG1 targeted miR-330-5p, leading to increased DCLK1 expression. This activated PI3K/AKT signaling and promoted cisplatin resistance (Ge et al., 2021). Additionally, Zhu et al. found that lncRNA FGD5-AS1 interacted with miR-142 to upregulate PD-L1 expression, which reduces cisplatin sensitivity (Zhu et al., 2021). Chen et al. found that lncRNA MALAT1 was highly expressed in the tissues of gemcitabine-resistant patients. MALAT1 upregulated PBOV1 level through sponge miR-27a-5p, thereby promoting gemcitabine resistance in NSCLC (Chen W. et al., 2022).

#### 3.2.2 LncRNAs related with radiotherapy in LC

Radiotherapy remains a key treatment modality for lung cancer. Over the past two decades, significant advancements in radiotherapy, such as stereotactic ablative radiotherapy (SABR) and intensity-modulated radiotherapy (IMRT), have improved treatment efficacy while minimizing radiation-induced damage to healthy tissues, ultimately enhancing patient survival and prognosis. Emerging evidence suggested that certain lncRNAs affected the radiosensitivity of lung cancer by directly regulating downstream gene expression, thereby contributing to tumor progression. For instance, Gao et al. demonstrated that lncRNA PCAT1 exerted an immunosuppressive effect and was associated with NSCLC invasion. The increase of PCAT1 expression was inversely correlated with immune cell infiltration in NSCLC tissues. Further investigation revealed that PCAT1 reduced radiosensitivity by activating SOX2 through modulation of the cGAS/STING signaling pathway (Gao et al., 2022).

Additionally, lncRNAs also act as competitive endogenous RNAs (ceRNAs) to modulate radiosensitivity in lung cancer. Ma et al. found that lncRNA protein tyrosine phosphatase receptor G-type antisense RNA 1 (lncRNA PTPRG-AS1) reduced NSCLC radiosensitivity by sponging miR-200c-3p, leading to upregulation of TCF4 expression (Ma et al., 2020). Jiang et al. showed that lncRNA cytoskeleton regulator (lncRNA CYTOR) was upregulated in NSCLC and reduced radiosensitivity by downregulating miR-206 and activating prothymosin α (Jiang et al., 2021). Moreover, in a research by Wang et al. it was found that overexpression of lncRNA hepatocyte nuclear factor 1α antisense RNA 1 (HNF1A-AS1) sponged miR-92a-3p, leading to upregulation of MAP2K4. This, in turn, activated JNK phosphorylation and promoted radioresistance in NSCLC (Wang et al., 2021).

Overall, lncRNAs modulate NSCLC radiosensitivity through diverse mechanisms, including gene expression regulation and signaling pathway modulation. However, Given the complexity and functional diversity of lncRNAs, further research is needed to elucidate their regulatory networks and explore their potential as biomarkers or therapeutic targets for clinical application.

#### 3.2.3 LncRNAs as diagnostic and prognostic biomarkers in LC

Advances in detection technologies have led to the identification of numerous lncRNAs as potential diagnostic and prognostic biomarkers in lung cancer. For example, Weber et al. found that MALAT1 was readily detectable in the peripheral blood of NSCLC patients compared to healthy controls, suggesting its potential as a diagnostic biomarker (Weber et al., 2013). Beyond diagnosis, MALAT1 is also linked to patient prognosis. Schmidt et al. reported that high MALAT1 expression correlated with poor prognosis in NSCLC, and when combined with thymosin β4, it served as an independent prognostic indicator for early-stage disease (Schmidt et al., 2011). In addition, lncRNA NEAT1 (Jen et al., 2017) and TUG1 (Da et al., 2021) were highly expressed in NSCLC tissues, which were associated with the poor prognosis of NSCLC patients, highlighting their potential as prognostic markers. Additional insights into the role of lncRNAs in lung cancer progression are provided in the work of Ge et al. (2024).

### 3.3 CircRNAs in LC

Studies have shown that circRNA is involved in multiple cellular biochemical processes of NSCLC, including proliferation, differentiation, metastasis, apoptosis and ferroptosis, indicating that circRNAs plays a vital role in NSCLC (Shanshan et al., 2021; Lu et al., 2019; Shang et al., 2019). In addition, circRNAs could induce tumor drug resistance through a variety of methods, including inhibiting cancer cell apoptosis, enhancing autophagy, accelerating drug excretion from cells, promoting DNA damage repair, and maintaining the characteristics of tumor stem cells (Fan et al., 2022; Wang et al., 2020; Huang et al., 2019; Xie H. et al., 2022; Zhong et al., 2021).

#### 3.3.1 CircRNAs mediating specific drug resistance of NSCLC

Emerging studies have shown that circRNAs could contribute to drug resistance in NSCLC, affecting agents such as paclitaxel (PTX), docetaxel (DTX), cisplatin, pemetrexed, gemcitabine, and osimertinib. (1) Regarding PTX resistance, Guo et al. demonstrated that circ_0011292 promoted PTX resistance in NSCLC by modulating the miR-379-5p/TRIM65 axis (Guo et al., 2022). Similarly, Xia et al. proved that overexpression of hsa_circ_0003489 drove PTX resistance by modulating miR-98-5p/IGF2 (Xia and Wang, 2023). These findings suggested that targeting circ_0011292 and circ_0002874 could be a potential therapeutic strategy to reverse PTX resistance. (2) Regarding DTX resistance, Zhang et al. reported that circ_0003998 reduced drug sensitivity of lung cancer cells to DTX by modulating the miR-136-5p/CORO1C axis (Zhang W. et al., 2021). In addition, Du et al. showed that high levels of circ_0014130 led to DTX resistance by regulating the miR-545-3p/YAP1 axis (Du et al., 2021). (3) For cisplatin resistance, He et al. identified that hsa_circ_0000190 promoted NSCLC cell resistance to cisplatin via the modulation of the miR-1253/IL-6 axis (He et al., 2024). Conversely, Song et al. found that circANKRD28 suppressed cisplatin resistance in NSCLC through the miR-221-3p/SOCS3 axis (Song et al., 2023). (4) For pemetrexed resistance, Zheng et al. manifested that circ_PVT1 enhanced resistance to both cisplatin and pemetrexed via the miR-145-5p/ABCC1 axis (Zheng and Xu, 2020). Mao et al. pointed out that circ CDR1-AS was highly expressed in lung cancer and promoted pemetrexed and CDDP chemotherapy resistance by regulating the EGFR/PI3K signaling pathway (Mao and Xu, 2020). (5) In gemcitabine resistance, Du et al. indicated that hsa_circ_0125356 acted as a driver of gemcitabine resistance via the miR-582-5p/FGF9 axis and WNT pathway (Du et al., 2025). In addition, Yu et al. pointed out that loss of circ_0092367 induced invasive EMT characteristics and gemcitabine resistance in NSCLC through regulating the miR-1206/ESRP1 axis (Yu S. et al., 2021). (6) In crizotinib resistance, Pan et al. showed that circRBM33 promotes resistance by regulating the DNMT1/IL-6 axis (Pan et al., 2023). Tang et al. manifested that circ_PPAPDC1A enhanced osimertinib resistance by sponging miR-30a-3p and activating the IGF1R pathway (Tang et al., 2024). Additional details on circRNA-mediated drug resistance in NSCLC are summarized in Table 2.

**TABLE 2:** CricRNA-mediated NSCLC-related specific resistance.

Drugs	CircRNAs	Targets	Effects	References
Paclitaxel	Hsa_circ_0003489	miR-98-5p/IGF2	Contributes resistance	Xia and Wang (2023)
	Circ_0058608	GBP1	Contributes resistance	Xuan et al. (2022)
	Circ_0000376	miR-1298-5p/KPNA4 axis	Contributes resistance	Hu et al. (2023)
	CircPIM3	miR-338-3p/TNFAIP8 axis	Contributes resistance	Du et al. (2022)
	Hsa_circ_0092887	miR-490-5p/UBE2T	Contributes resistance	Wang et al. (2022b)
	circ_0076305	miR-936/TMPRSS4 axis	Contributes resistance	Su et al. (2024)
Cisplatin	CircRNA_103615	ABCB1	Contributes resistance	Liang et al. (2021)
	CircRNA_100565	miR-337-3p/ADAM28 axis	Contributes resistance	Zhong et al. (2021)
	CircVMP1	miR-524-5p-METTL3/SOX2 axis	Contributes resistance	Xie et al. (2022a)
	Circ-CUL2	microRNA-888-5p/RB1CC1 axis	Enhance sensitivity	Chen et al. (2022c)
	Hsa_circ_0000190	miR-1253/IL-6 axis	Contributes resistance	He et al. (2024)
	Circ_PIP5K1A	ROCK1	Contributes resistance	Feng et al. (2021)
	CircPTK2	miR-942/TRIM16 axis	Enhance sensitivity	Wang et al. (2022c)
	Hsa_circ_0068252	miR-1304-5p/PD-L1 axis	Contributes resistance	Li et al. (2022c)
	circANKRD28	miR-221-3 p/SOCS3 axis	Enhance sensitivity	Song et al. (2023)
	Hsa_circ_0006006	-	Contributes resistance	Ding et al. (2025)
	Circ_0110498	miR-1287-5p/RBBP4 axis	Contributes resistance	Hao et al. (2023)
	Circ-ANXA7	CCND1	Contributes resistance	Yao et al. (2023)
	Circ_0076305	ABCC1	Contributes resistance	Wang et al. (2023)
	CircRNA B	miR-25-3p/BARX2 axis	Enhance sensitivity	Liu et al. (2023b)
Gefitinib	CircKIF20B	miR-615-3p/MEF2A axis	Enhance sensitivity	Wei et al. (2023)
	Circ_0001786	miR-34b-5p/SRSF1	Contributes resistance	Ouyang et al. (2024)
	Circ_0014235	miR-146b-5p/YAP/PD-L1 pathway	Contributes resistance	Niu et al. (2021)
	Circ_0001658	miR-409-3p/TWIST1 axis	Contributes resistance	Yu et al. (2021b)
	Circ_0091537	miR-520h/YAP1 axis	Contributes resistance	Qu and Ma (2023)
	CircSETD3	FXR1/ECT2	Contributes resistance	Wen et al. (2023)
Docetaxel	Circ_0003998	miR-136-5p/CORO1C axis	Contributes resistance	Zhang et al. (2021a)
	Circ_0014130	miR-545-3p/YAP1 axis	Contributes resistance	Du et al. (2021)
Osimertinib	CircRBM33	DNMT1/IL-6 axis	Contributes resistance	Pan et al. (2023)
	Circ_PPAPDC1A	miR-30a-3p/IGF1R	Contributes resistance	Tang et al. (2024)
	circSPECC1	-	Contributes resistance	Hao et al. (2024)
	CircMYBL1	-	Enhance sensitivity	Li et al. (2024c)
gemcitabine	Hsa_circ_0125356	WNT	Contributes resistance	Du et al. (2025)
	Circ_0092367	MiR-1206-ESRP1 axis	Enhance sensitivity	Yu et al. (2021a)

#### 3.3.2 CircRNAs as therapeutic targets to overcome drug resistance in NSCLC

As discussed, CircRNAs play a critical role in drug resistance in lung cancer and thus represent promising therapeutic targets. Current research primarily focuses on suppressing circRNA expression to restore the sensitivity of NSCLC cells to chemotherapy. For example, Zhang et al. demonstrated that shRNA-mediated knockdown of circ_0072088 significantly reduced cisplatin resistance in NSCLC cells (Zhang L. et al., 2022). Similarly, silencing circ_0004015 via siRNA and shRNA suppressed cisplatin resistance in NSCLC (Li Y. et al., 2022). Zheng et al. further demonstrated that inhibiting hsa_circ_0074027 reduced chemotherapy resistance by modulating the miR-379-5p/IGF1 signaling axis (Zheng et al., 2021). Additional insights into circRNAs as therapeutic targets in overcoming NSCLC drug resistance are detailed in the study by Yan et al. (2022).

## 4 Biologic functions and molecular mechanism of non-coding RNAs-medicated autophagy in lung cancer

During the progression of lung cancer, abnormally regulated ncRNAs and autophagy are important factors that regulate related signaling pathways at the transcriptional, post-transcriptional and post-translational levels, thereby changing various malignant behaviors and therapeutic responses of lung cancer.

### 4.1 MiRNAs-medicated autophagy promoted the progression of lung cancer

#### 4.1.1 MiRNAs promoted autophagy to promoted lung cancer

**FIGURE 3:**
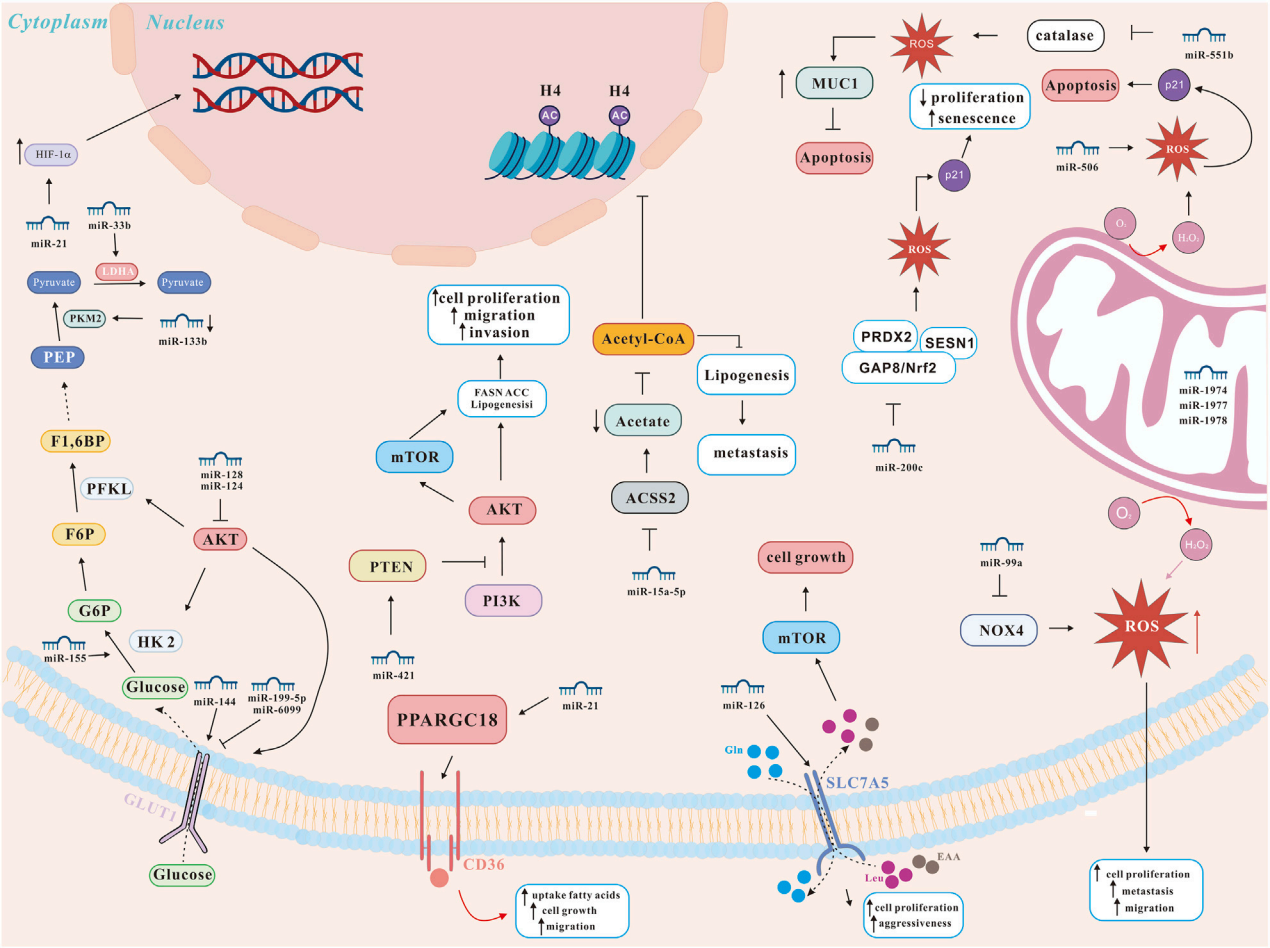
miRNA network regulating lung cancer metabolism (modified from (Carrà et al., 2024)). ACSS2, AMP-activated protein kinase-mediated acetyl-CoA synthetase 2; AKT, protein kinase B; GLUT, glucose transporters; HIF-1α, hypoxia-inducible factor 1 alpha; HK2, hexokinase 2; LDHA, lactate dehydrogenase A; mTOR, mechanistic target of rapamycin; MUC1, transmembrane glycoprotein mucin 1; NOX4, NADPH oxidase 4; Nrf2, nuclear factor erythroid 2-related factor 2; PFKL, phosphofructokinase, liver type; PKM2, pyruvate kinase isoenzyme M2; PI3K, phosphatidylinositol 3-kinase; PPARGC1B, peroxisome proliferator-activated receptor gamma coactivator 1-beta; PRDX2, peroxiredoxin 2; PTEN, phosphatase and tensin homolog; ROS, reactive oxygen species; SESN1, sestrin 1.

Many studies have explored the molecular mechanism of lung cancer, many of which have confirmed the key role of miRNAs and autophagy in lung cancer progression and drug resistance (Li S. et al., 2018; Liu J. et al., 2018) (Figure 3). On the one hand, miRNAs can act as pro-survival factors for lung cancer by regulating autophagy. For instance, miRNAs promoted lung cancer tumorigenesis by promoting autophagy. Li et al. reported that miRNA-21 promoted the proliferation, migration and invasion of NSCLC cells through reducing autophagy via AMPK/ULK1 signaling pathway (Li S. et al., 2018). Liang et al. reported that miR-18a-5p promoted autophagy and carcinogenesis of lung cancer by directly targeting IRF2 (Liang et al., 2017).

#### 4.1.2 MiRNAs inhibited autophagy to promoted lung cancer

In addition, miRNAs could also promote lung cancer by inhibiting autophagy. For instance, Ju et al. reported that miR-210 directly targeted ATG7 to reduce the level of autophagy and promoted the proliferation of lung cancer cells (Ju et al., 2019). Tang et al. reported that miR-3127-5p promoted STAT3 phosphorylation through suppressing autophagy and upregulated PD-L1 resulting in chemoresistance in NSCLC (Tang et al., 2018). Furthermore, Bao et al. found that miR-487b-5p promoted proliferation and migration of temozolomide-resistance lung cancer cells through Lamp2-medicated autophagy (Bao et al., 2016). The above results indicated that miRNA promoted the progression of lung cancer by regulating autophagy.

### 4.2 MiRNAs-medicated autophagy inhibited the progression of lung cancer

#### 4.2.1 MiRNAs promoted autophagy to inhibited lung cancer

On the other hand, miRNAs can also act as pro-death factors in lung cancer by regulating autophagy such as miRNA-106a, a member of the miR-17 family, has been found to be abnormally expressed in a variety of cancers (Pan et al., 2016), and is closely related to the occurrence, development and prognosis of cancer (Zhao et al., 2019). Han et al. reported that upregulation of miR-106a promoted metastasis by targeting tumor protein 53-induced nuclear protein 1 (TP53INP1)-mediated metastasis progression, including cell migration, autophagy-dependent death, and epithelial-mesenchymal transition (EMT) (Han et al., 2021). Guo et al. reported that miR-384 inhibited cell proliferation and promoted cell apoptosis and autophagy in NSCLC cells through down-regulating collagen α-1(X) chain (COL10A1) levels (Guo et al., 2019). Chen et al. showed that miR-144 targeted p53-induced glycolysis and apoptosis regulator (TIGAR) and increased autophagy, thereby promoting apoptosis and inhibiting lung cancer cell proliferation (Chen et al., 2015). In addition, Ye et al. suggested that miR-138 suppressed the proliferation, metastasis and autophagy of NSCLC cells by targeting Sirt1 (Ye et al., 2017).

#### 4.2.2 MiRNAs inhibited autophagy to inhibited lung cancer

In a research by Hua et al., it was demonstrated that miR-1 overexpression improved cisplatin chemosensitivity of NSCLC cells by inhibiting ATG3-mediated autophagy (Hua et al., 2018). In an academic study conducted by He et al. it was reported that miR-26 promoted apoptosis and suppressed autophagy in NSCLC cells by inhibiting TGF-β1-JNK signaling pathway (He et al., 2019). Furthermore, both miR-142-3p and miRNA-153-3p promoted the proliferation of lung cancer through inhibiting the autophagy pathway (Zhang W. et al., 2019; Meng et al., 2024). More about the relationship between miRNAs-medicated autophagy in lung cancer is shown in Table 3.

**TABLE 3:** Autophagy-related miRNAs in lung cancer.

miRNAs	Anti-/Proautophagy	Pro/Antitumor	Possible mechanisms	References
miR-384	Proautophagy	Antitumor	miR-384 downregulated COL10A1 levels, subsequently inhibited cell proliferation and promoted cell apoptosis and autophagy in NSCLC cells	Guo et al. (2019)
miR-21	Proautophagy	Pro-tumor	MiR-21 regulated autophagy through AMPK/Ulk1 signaling pathway and promoted proliferation, migration and invasion of NSCLC	Li et al. (2018a)
miR-181	Proautophagy	Antitumor	MiR-181 regulated cisplatin-resistant NSCLC by down-regulating autophagy through PTEN/PI3K/AKT pathway	Liu et al. (2018a)
miR-18a-5p	Proautophagy	Pro-tumor	MiR-18a-5p promoted autophagy and carcinogenesis of lung cancer by directly targeting IRF2	Liang et al. (2017)
miR-106a	Proautophagy	Antitumor	MiR-106a enhanced the sensitivity of lung cancer cells to Src inhibitors by targeting autophagy	Rothschild et al. (2017)
miR-16	Proautophagy	Antitumor	MiR-16 mimics induced epithelial-mesenchymal transition in NSCLC cells by activating autophagy	Wang et al. (2017)
miR-17	Proautophagy	Pro-tumor	BLACAT1 promoted ATG7 expression through miR-17, and facilitated autophagy and promoted chemoresistance of NSCLC cells through miR-17/ATG7	Huang et al. (2018)
miR-138	Proautophagy	Antitumor	miR-138 suppressed the proliferation, metastasis and autophagy of NSCLC by targeting Sirt1	Ye et al. (2017)
miR-144	Proautophagy	Antitumor	MiR-144 inhibited proliferation and induces apoptosis and autophagy in lung cancer cells by targeting TIGAR	Chen et al. (2015)
miR-124	Proautophagy	Antitumor	MiR-124 repressed p62 and beclin-1 selectively in KM cells leading to apoptosis and inhibiting clonogenic growth	Mehta et al. (2017)
miR-210	Antiautophagy	Pro-tumor	Upregulation of miR-210 resulted in decrease in the expression of ATG7, LC3-II/LC3-I and Beclin-1, while transfection of miR-210 inhibitors increased the expression of the mentioned targets	Ju et al. (2019)
miR-23a	Antiautophagy	Antitumor	Pristimerin enhanced the effect of cisplatin by inhibiting the miR-23a/Akt/GSK3β signaling pathway and suppressing autophagy in lung cancer cells	Zhang et al. (2019b)
miR-1	Antiautophagy	Antitumor	MiR-1 overexpression improved cisplatin sensitivity of NSCLC cells by inhibiting ATG3-mediated autophagy	Hua et al. (2018)
miR-3127-5p	Antiautophagy	Pro-tumor	MiR-3127-5p promoted STAT3 phosphorylation through suppressing autophagy and upregulated PD-L1 inducing chemoresistance in NSCLC	Tang et al. (2018)
miR-195-5p	Antiautophagy	Antitumor	Hsa-miR-195-5p promoted apoptosis, repressed proliferation, and autophagy via E2F7/CEP55 and reduced gemcitabine resistance in lung adenocarcinoma	Fu et al. (2023)
miR-487b-5p	Antiautophagy	Pro-tumor	MiR-487b-5p regulated temozolomide resistance of lung cancer cells through regulating LAMP2-medicated autophagy	Bao et al. (2016)
miR-142-3p	Antiautophagy	Antitumor	MiR-142-3p regulated starvation-induced autophagy of NSCLC cells by directly downregulating HMGB1 and subsequently activating the PI3K/Akt/mTOR pathway	Meng et al. (2024)
miR-26	Antiautophagy	Antitumor	miR-26 induced apoptosis and inhibited autophagy in human NSCLC cells through the TGF-1-JNK signaling pathway	He et al. (2019)
miR-146a-5p	Antiautophagy	Antitumor	MiR-146a-5p increased chemosensitivity of NSCLC to cisplatin by targeting Atg12 to inhibit autophagy	Yuwen et al. (2017)

### 4.3 LncRNAs-medicated autophagy promoted the progression of lung cancer

#### 4.3.1 LncRNAs promoted autophagy to promoted lung cancer

Numerous studies have also highlighted the critical roles of both autophagy and lncRNAs in lung cancer progression. Like autophagy-related miRNAs, autophagy-related lncRNAs can function as both promoters and suppressors of lung cancer. For instance, Wang et al. demonstrated that lncRNA MSTO2P enhanced lung cancer cell proliferation and autophagy by upregulating EZH2 expression (Wang et al., 2019). Hu et al. found that lncRNA MITA1 increased gefitinib resistance in lung cancer cells through autophagy induction (Hu et al., 2021). Similarly, Xin et al. demonstrated that lncRNA MALAT-1 facilitated autophagy and NSCLC growth by recruiting E2F1 to upregulate RAD51 expression (Xin et al., 2023). Furthermore, Furthermore, Xie and Hu et al. reported that lncRNA NEAT1 enhanced NSCLC drug resistance and suppressed apoptosis by inducing autophagy (Xie Y. et al., 2022; Hu et al., 2024). Song et al. revealed that lncRNA NFYC-AS1 promoted the proliferation of lung cancer by regulating autophagy and apoptosis (Song et al., 2021). In addition, lncRNAs such as, TEX41 (Li R. et al., 2023), BLACAT1 (Huang et al., 2018), Linc00673-V3 (Ni et al., 2024) promoted lung cancer cell growth or mediated resistance to chemotherapeutic drugs via activating autophagy.

#### 4.3.2 LncRNAs inhibited autophagy to promoted lung cancer

Wu et al. reported that LINC01279 was highly expressed in lung cancer cells, and inhibiting the function of LINC01279 reduced the growth of xenograft tumors of NSCLC cells. Further studies have found that knockdown of LINC01279 or SIN3A activated autophagy and apoptosis of NSCLC cells. Therefore, LINC01279 promoted the occurrence and development of lung cancer by regulating FAK and SIN3A to inhibit autophagy in lung cancer cells (Wu et al., 2024). In a research by Li et al., it was found that lncRNA KTN1-AS1 was upregulated in NSCLC tissues and positively correlated with poor prognosis. In addition, knockdown of KTN1-AS1 inhibited the growth and proliferation of NSCLC cells and increased apoptosis. Further studies have found that KTN1-AS1 regulated the expression of miR-130a-5p target gene PDPK1 in NSCLC cells to inhibit autophagy in lung cancer cells. Therefore, KTN1-AS1 inhibited autophagy in lung cancer cells through miR-130a-5p/PDPK1 signaling pathway (Li et al., 2020). Furthermore, Yang et al. reported that LINC00941 was a diagnostic biomarker for lung adenocarcinoma and promoted tumorigenesis through inhibiting cell autophagy (Yang et al., 2024). The above three lncRNAs promoted lung cancer cells growth of by inhibiting autophagy pathway.

### 4.4 LncRNAs-medicated autophagy inhibited the progression of lung cancer

#### 4.4.1 LncRNAs promoted autophagy to inhibited lung cancer

Additionally, lncRNAs-medicated autophagy can act as pro-death factors in lung cancer. For instance, Zhang et al. demonstrated that the expression of lncRNA PANDAR and autophagy-related gene BECN1 was downregulated in lung cancer. Further studies showed that the high expression of PANDAR increased the expression level of BECN1, promoted autophagy and apoptosis, and inhibited the proliferation of NSCLC. Therefore, lncRNA PANDAR was a tumor suppressor that inhibited the proliferation of NSCLC cells by up-regulating the activation of autophagy and apoptosis pathways (Zhang L. et al., 2020). Another related study showed that the expression of lncRNA-XIST in non-small cell lung cancer tissues was significantly higher than that in adjacent normal tissues. In addition, knockdown of lncRNA-XIST reduced basal autophagy and autophagic flux in NSCLC cells. Further studies found that knockdown of XIST weakened autophagy-dependent NSCLC chemotherapy resistance. Therefore, overexpression of lncRNA XIST has been linked to increased cisplatin resistance in chemotherapy, mediated through autophagy activation via the miR-17/ATG7 signaling pathway (Sun et al., 2017).

#### 4.4.2 LncRNAs inhibited autophagy to inhibited lung cancer

LncRNAs could also inhibited lung cancer by inhibiting autophagy such as Gupta et al. reported that targeting lncRNA DLX6-AS1 enhanced miR-16 activity, inducing autophagy and apoptosis, while regulating BMI1 through miR-16 sponging, thereby inhibiting lung cancer growth (Gupta et al., 2023). Li et al. found that lncRNA CASC2 inhibited autophagy and promoted apoptosis in NSCLC cells via regulating the miR-214/TRIM16 axis (Li Q. et al., 2018). Furthermore, lncRNAs such as MEG3 (Xia et al., 2018), ACTA2-AS1 (Liu et al., 2022), NBAT1 (Zheng et al., 2018), HOTAIR (Yang et al., 2018), have been implicated to suppressing lung cancer cell growth or mediating resistance to chemotherapeutic drugs via inhibiting autophagy mechanisms. More about the relationship between lncRNAs-medicated autophagy in lung cancer is shown in Table 4.

**TABLE 4:** Autophagy-related LncRNAs in lung cancer.

LncRNAs	Anti-/Proautophagy	Pro/Antitumor	Possible mechanisms	References
MSTO2P	Proautophagy	Pro-tumor	Promoting lung cancer cell proliferation and autophagy by up-regulating EZH2	Wang et al. (2019)
MITA1	Proautophagy	Pro-tumor	Inducing autophagy and suppressing apoptosis	Hu et al. (2021)
MALAT1	Proautophagy	Pro-tumor	Recruiting E2F1 to upregulate RAD51 expression	Xin et al. (2023)
NEAT1	Proautophagy	Pro-tumor	Targeting the mir-379-3p/HIF1A pathway	Xie et al. (2022b), Hu et al. (2024)
NFYC-AS1	Proautophagy	Pro-tumor	Including autophagy and downregulating oncoproteins such as MET and c-Myc	Song et al. (2021)
RNALINC01279	Antiautophagy	Pro-tumor	Regulating FAK and SIN3A	Wu et al. (2024)
TEX41	Proautophagy	Pro-tumor	Increasing Runx2 expression	Li et al. (2023b)
BLACAT1	Proautophagy	Pro-tumor	Targeting the miR-17/ATG7 signaling pathway	Huang et al. (2018)
Linc00673-V3	Proautophagy	Pro-tumor	Promoting Smad3-mediated *LC3B* transcription	Ni et al. (2024)
KTN1-AS1	Antiautophagy	Pro-tumor	Sponging of miR-130a-5p and activation of PDPK1	Li et al. (2020)
LINC00941	Antiautophagy	Pro-tumor	Targeting PI3K/AKT/mTOR pathway possibly	Yang et al. (2024)
PANDAR	Proautophagy	Antitumor	Regulating autophagy and apoptosis pathways	Zhang et al. (2020b)
XIST	Proautophagy	Antitumor	Inducing autophagy by the miR-17/ATG7 signaling pathway	Sun et al. (2017)
DLX6-AS1	Antiautophagy	Antitumor	Regulating BMI1 through sponging miR-16 expression	Gupta et al. (2023)
CASC2	Antiautophagy	Antitumor	Regulating the miR-214/TRIM16 axis	Li et al. (2018b)
MEG3	Antiautophagy	Antitumor	Inhibiting autophagy	Xia et al. (2018)
ACTA2-AS1	Antiautophagy	Antitumor	Suppressing TSC2 expressing by recruiting EZH2 to TSC2 gene promoter	Liu et al. (2022)
NBAT1	Antiautophagy	Antitumor	Inhibiting autophagy via suppression of ATG7	Zheng et al. (2018)
HOTAIR	Antiautophagy	Antitumor	Suppressing phosphorylation of ULK1	Yang et al. (2018)

### 4.5 CircRNAs-medicated autophagy promoted the progression of lung cancer

#### 4.5.1 CircRNAs promoted autophagy to promoted lung cancer

Unlike traditional linear RNAs, circular RNAs (circRNAs) have a closed-loop structure, making them resistant to RNA exonucleases. This unique structure ensures their stability and resistance to degradation. Functionally, circRNAs are abundant in miRNAs-binding sites and act as miRNAs sponges within cells. This interaction alleviates the suppressive effects of miRNAs on their target genes, thereby enhancing target gene expression. Studies have shown that autophagy and circRNAs were crucial in lung cancer progression. Notably, some circRNAs regulate lung cancer cell growth by enhancing autophagy. For instance, Wei et al. demonstrated that circular RNA circ-FOXM1 promoted autophagy and drived NSCLC progression by suppressing miR-149-5p expression and upregulating ATG5 levels (Wei et al., 2021). Zhong et al. identified that circRNA_100565 as a contributor to cisplatin resistance in NSCLC by promoting autophagy and suppressing apoptosis via the miR-337-3p/ADAM28 axis (Zhong et al., 2021). Likewise, Kong et al. reported that circular RNA hsa_circ_0085131 enhanced cisplatin resistance in NSCLC cells by stimulating autophagy (Kong, 2020). Additionally, circular RNA Hsa_circ_0010235 (Zhang F. et al., 2021) and circular RNA Hsa_circ_0096157 (Lu et al., 2024) have been reported to facilitate NSCLC growth through autophagy activation.

#### 4.5.2 CircRNAs inhibited autophagy to promoted lung cancer

Yang and Zhang identified that circular RNA hsa_circ_0020123 suppressed autophagy by sponging specific miRNAs, thereby promoting lung cancer cell growth (Zhang H. et al., 2022; Yang et al., 2022). Similarly, Guan et al. demonstrated that circular RNA circ_0003028 inhibited autophagy and drived tumorigenesis in NSCLC by modulating GOT2 expression via miR-1298-5p (Guan et al., 2021). Additionally, circular RNA *circHIPK3* and circular RNA EHD2 have been shown to facilitate NSCLC growth by inhibiting autophagy (Chen X. et al., 2019; Zhang F. et al., 2022). For further details on circRNAs-medicated autophagy in lung cancer, refer to Table 5.

**TABLE 5:** Autophagy-related CircRNAs in lung cancer.

CircRNAs	Anti-/Proautophagy	Pro/Antitumor	Possible mechanisms	References
circ-FOXM1	Proautophagy	Pro-tumor	Regulating the miR-149-5p/ATG5 axis	Wei et al. (2021)
circRNA_100565	Proautophagy	Pro-tumor	Regulating proliferation, apoptosis and autophagy via miR-337-3p/ADAM28 axis	Zhong et al. (2021)
hsa_circ_0085131	Proautophagy	Pro-tumor	Targeting miR‐654‐5p to upregulate ATG7	Kong (2020)
hsa_circ_0010235	Proautophagy	Pro-tumor	Modulating miR-433-3p/TIPRL axis	Zhang et al. (2021b)
hsa_circ_0096157	Proautophagy	Pro-tumor	Weakening the Nrf2/ARE signaling pathway	Lu et al. (2024)
circ_0020123	Antiautophagy	Pro-tumor	Upregulating IRF4 through eliminating miR-193a-3p-mediated suppression of IRF4	Zhang et al. (2022b), Yang et al. (2022)
circ_0003028	Antiautophagy	Pro-tumor	Regulating GOT2 via miR-1298-5p	Guan et al. (2021)
circHIPK3	Antiautophagy	Pro-tumor	Targeting miR124-3p-STAT3-PRKAA/AMPKα signaling	Chen et al. (2019d)
circEHD2	Antiautophagy	Pro-tumor	Crosstalking with microRNA-3186-3p and forkhead box K1	Zhang et al. (2022c)

## 5 Limitations of the study

Notably, autophagy and ncRNAs (miRNAs, lncRNAs, circRNAs) are pivotal in lung cancer development and progression. These molecules can function as cancer promoters, driving lung cancer growth and chemoresistance, or as tumor suppressors, inhibiting tumor progression. However, several limitations persist in understanding and analyzing the roles of autophagy and ncRNAs in lung cancer: 1) Individual Variability: the heterogeneity among lung cancer patients and the complexity of the tumor microenvironment result in substantial individual differences in ncRNAs expression levels and autophagy activity. These variations complicate the accurate assessment of their roles in lung cancer progression. 2) Unclear mechanisms: while ncRNAs significantly influence lung cancer by modulating autophagy pathways, the precise mechanisms of many ncRNAs remain poorly understood. Moreover, it is often unclear whether ncRNA-based therapies act solely through autophagy, requiring further research. 3) Lack of specificity: current ncRNA-based drugs and autophagy modulators often lack disease specificity, potentially causing collateral damage to normal tissues and limiting their clinical application in lung cancer treatment. 4) Drug delivery challenges: delivering ncRNAs and autophagy modulators faces significant barriers, including instability, low specificity, and limited tumor-targeting efficiency. These issues reduce therapeutic efficacy and increase the risk of systemic side effects. 5) Insufficient Monitoring Tools: the dual role of autophagy and ncRNAs in lung cancer (both tumor-suppressing and tumor-promoting) underscores the need for reliable real-time monitoring tools. Without these, dynamic changes in autophagy and ncRNAs during treatment may lead to suboptimal outcomes or adverse effects.

## 6 Future directions

To address the limitations mentioned above, the following strategies may offer valuable insights:

(1) Identifying highly specific biomarkers: Ideally, highly specific markers can include ncRNAs expression profiles and detectable autophagy-related genes or proteins (such as in blood or sputum). These markers could provide accurate insights into dynamic changes during lung cancer progression. Monitoring these markers can reduce invasive diagnostic procedures, lower economic burdens, track tumor progression and treatment response, and guide personalized therapeutic strategies. It is believed that in the future, with the continuous development of high-throughput screening technology, bioinformatics prediction, luciferase reporter gene assay, mass spectrometry analysis technology, more and more high-specificity will be reported.
(2) Designing Targeted ncRNA Therapeutics: The specificity of ncRNA modulators is critical for minimizing off-target effects and improving therapeutic efficacy. By focusing on autophagy-related pathways (e.g., Beclin1 or mTORC1), ncRNA mimics or inhibitors could be tailored for specific tumor stages (characterized by excessive autophagy activation or inhibition). Combining ncRNA-based therapies with traditional treatments, such as chemotherapy or immunotherapy, may further amplify therapeutic benefits and reduce adverse drug reactions.
(3) Implementing real-time monitoring: Real-time systems to monitor ncRNA expression, autophagy activity, and drug concentrations during treatment are critical, given the dual role of autophagy in lung cancer. Advanced imaging techniques, like nanoparticle-based probes, could track autophagic flux and ncRNAs dynamics *in vivo*, preventing excessive or insufficient drug modulation that could worsen the disease or lead to drug resistance.
(4) Using targeted drug delivery systems: Efficient delivery of ncRNA-based therapies remains a key challenge. Utilizing targeted delivery methods, such as lipid nanoparticles or exosomes, could enhance the stability and tumor specificity of these drugs, significantly reducing side effects and improving therapeutic outcomes.
(5) Focus on the subcellular distribution of ncRNAs: The factors driving the subcellular distribution of circRNAs and the biogenesis and transport of ncRNAs (including extracellular transport and degradation) were investigated by using live cell imaging techniques.

## 7 Conclusion

In general, existing studies have shown that ncRNA and autophagy play an important role in the occurrence and progression of lung cancer, which makes them potential therapeutic targets. However, the specific mechanism between ncRNA and autophagy still needs further study. The development of ncRNA-based therapeutic strategies, especially ncRNAs that regulate autophagy pathways, may provide new ideas and methods for the treatment of lung cancer. In addition, since current autophagy modulators and ncRNA drugs often lack sufficient targeting and specificity, improving the targeting and efficacy of these drugs will be an important topic in future research.
